# Biological testing of chitosan‐collagen‐based porous scaffolds loaded with PLGA/Triamcinolone microspheres for ameliorating endoscopic dissection‐related stenosis in oesophagus

**DOI:** 10.1111/cpr.13004

**Published:** 2021-02-04

**Authors:** Wenkai Ni, Shengli Lin, Saiyan Bian, Mingbing Xiao, Yongjun Wang, Yumin Yang, Cuihua Lu, Wenjie Zheng, Pinghong Zhou

**Affiliations:** ^1^ Endoscopy Center and Endoscopy Research Institute Zhongshan Hospital Fudan University Shanghai China; ^2^ Department of Gastroenterology Affiliated Hospital of Nantong University Nantong China; ^3^ Medical College Nantong University Nantong China; ^4^ Research Center of Clinical Medicine Affiliated Hospital of Nantong University Nantong China; ^5^ Key Laboratory of Neuroregeneration of Jiangsu and Ministry of Education Co‐innovation Center of Neuroregeneration Nantong University Nantong China

**Keywords:** ESD, microsphere, scaffold, stricture, triamcinolone acetonide

## Abstract

**Objectives:**

Endoscopic submucosal dissection (ESD), a preferential approach for early oesophageal neoplasms, inevitably results in oesophageal strictures in patients. Clinical use of glucocorticoids through submucosal injection is beneficial for inhibiting oesophageal stricture following injury; however, it also has limitations, such as dose loss and perforation. Hence, alternatives to glucocorticoid therapy should be developed.

**Methods:**

A novel porous composite scaffold, ChCo‐TAMS, composed of chitosan, collagen‐I and triamcinolone acetonide (TA) loaded into poly (lactic‐co‐glycolic) acid (PLGA) microspheres (TAMS), was successfully constructed and subjected to biological testing to ameliorate oesophageal ESD‐related stenosis.

**Results:**

The synthesized biomaterials displayed unique properties in inhibiting the activation of macrophages, chemokine‐mediated cell recruitment and fibrogenesis of fibroblasts. Further application of the scaffolds in the rat dermal defect and porcine oesophageal ESD model showed that these novel scaffolds played a robust role in inhibiting wound contracture and oesophageal ESD strictures.

**Conclusions:**

The developed composite scaffolds provide a promising clinical medical device for the prevention of post‐operative oesophageal stricture.

## Graphical abstract

The composite porous scaffold ChCo‐TAMS containing chitosan, collagen and PLGA/Triamcinolone microspheres were synthesized and cross‐linked by genipin. In vitro assays demonstrated the inhibitory effects of the scaffolds upon macrophages activation and myofibroblasts transdifferentiation. The porcine model was established by performing circumferential ESD in oesophagus, and the post‐operative stricture was efficiently ameliorated by application of ChCo‐TAMS scaffolds.

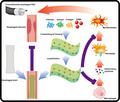

## 1 INTRODUCTION

With the development of endoscopic imaging techniques, including narrow band imaging and confocal laser endomicroscopy, the early diagnosis and management of oesophageal cancer are easier to achieve than ever before.^1^, ^2^ Endoscopic submucosal dissection (ESD) has been well established for the curative removal of early superficial oesophageal carcinoma.^3^ However, the resultant mucosal defect always results in severe stenosis, leading to dysphagia and poor quality of life.^4^ Cutting extension is generally regarded as the most important risk factor for post‐operative stenosis.^5^ Patients with semi‐circumferential oesophageal ESD are prone to oesophageal stricture at a rate of 70%‐90%.^6^ Hence, preventing post‐ESD stricture has become a widespread concern among endoscopists.

Glucocorticoids have been used as the preferred drug for oesophageal strictures because of their anti‐inflammatory and anti‐fibrotic effects.^7^ By submucosal injection, the drug at a low dose is released in situ for action.^8^ Clinically, a dose of 18‐62 mg of triamcinolone acetonide (TA) is administered to the submucosal tissue of the cutting edge or ulcer bed at 3, 7 and 10 days after ESD.^9^ Despite the remarkable effects of stricture inhibition, this procedure has certain disadvantages. For example, inevitable leakage of the drug weakens the therapeutic effects, improper injection to the muscle layer causes delayed perforation, and repeated endoscopic interventions reduce patient compliance.^10^ Therefore, alternatives for glucocorticoid delivery and sustained release need to be considered. Poly(lactic‐co‐glycolic) acid (PLGA) is a commonly used biodegradable polymer for drug loading and long‐term release.^11^ By forming microspheres (MS) that encapsulate TA, PLGA‐based delivery systems have shown promising advantages with a longer glucocorticoid duration and fewer side effects when tested in uveitis.^12^, ^13^ Thus, it is proposed that the system (PLGA/TA microspheres) can be applied to suppress post‐ESD oesophageal stricture.

The submucosal extracellular matrix (ECM) in the oesophagus is mainly composed of a mesh of glycosaminoglycans (GAGs) and protein fibres. Chitosan, a heteropolysaccharide derivative of chitin, shares high structural similarity with GAGs.^14^ Owing to its excellent biocompatibility, biodegradability and non‐toxicity, chitosan has been successfully applied in tissue engineering by constructing various types of scaffolds.^15^ It has also been reported to enhance the diffusion of glucocorticoids in the oesophageal epithelium.^16^ Another important component of the ECM that has been used for tissue repair and scar inhibition is collagen. Skin substitutes composed of collagen sponge and endothelial cells promote rapid vascularization during wound healing.^17^ More importantly, a recent study substantiates the efficacy of high‐density collagen patches in the inhibition of ESD‐related oesophageal stricture.^18^ Hence, constructing a composite porous 3D scaffold with incorporated chitosan, collagen and PLGA/TA microspheres might be a promising strategy to control ESD‐related oesophageal stricture by mimicking the natural fibrillary architecture of the ECM and reducing the local inflammatory and fibrotic processes.

In this study, a novel porous composite scaffold composed of chitosan, collagen and PLGA/TA microspheres (ChCo‐TAMS) was designed and constructed using the natural cross‐linker genipin.^19^ We determined the prolonged release of TA from PLGA/TA microspheres or ChCo‐TAMS to evaluate the drug delivery system under physiological pH conditions of the oesophagus. The properties of the composite scaffold, including biocompatibility, anti‐inflammation and anti‐fibrosis, were tested in vitro using various cell lines. To gain insight into its effect on inhibiting ESD‐related oesophageal strictures, the ChCo‐TAMS scaffold was transplanted into a rat dermal defect or porcine oesophageal ESD model, which showed satisfactory outcomes. Our results provide a potential therapeutic approach for the inhibition of ESD‐related oesophageal strictures.

## 2 MATERIALS AND METHODS

### 2.1 Preparation of PLGA/TA microspheres

PLGA/TA microspheres were prepared by solid‐in‐oil‐in‐water (s/o/w) double‐emulsion solvent evaporation. Briefly, 30 mg of TA (10% theoretical loading) and 270 mg of PLGA (18 kDa, 502 H, RESOMER) were dissolved in 500 μL methylene chloride, followed by homogenization at 10 000 rpm for 1.5 min to form a solid‐in‐oil (s/o) solution. Then, the products were slowly transferred to a 5% polyvinyl alcohol (PVA) (Sigma) solution with a moderate vortex (5000 rpm) for 2 minutes. The final s/o/w emulsion was rapidly transferred to a 0.5% PVA solution with constant rotation for 3 hours to fully evaporate the methylene chloride. The PLGA/TA microspheres were harvested by freeze‐drying and stored at −20°C before use.

### 2.2 Determination of encapsulation efficiency for the PLGA/TA microspheres

Five milligrams of PLGA/TA microspheres was dissolved in 20 mL acetonitrile, followed by high‐performance liquid chromatography (HPLC) to determine the TA content. Specifically, a C18 column (5 μm, 4.6 × 250 mm) was kept at 30°C. The mobile phase was a composite of methanol and water (70:30, v/v), and the flow rate was set at 1.0 mL/min. TA was detected at a wavelength of 240 nm. Drug loading was determined as the ratio of the drug in the microspheres to the mass of the microspheres, and encapsulation efficiency was calculated as the drug loading percentage of the theoretical loading.

### 2.3 Preparation of porous ChCo‐TAMS scaffold

Chitosan (Nantong fisheries Institute, China) and atelocollagen‐I extracted from bovine tendon (Saining Biotechnology, China) were dissolved in 0.2 M acetic acid at final concentration of 0.5% (w/v) each. Then, 0.1% genipin with or without the prepared PLGA/TA microspheres (weight ratio, chitosan/collagen: TA microspheres = 1:2) was added to the composite solution. Composite scaffolds were fabricated under different conditions, resulting in distinct properties. For chitosan/collagen (ChCo) scaffolds without the addition of PLGA/TA microspheres, the mixed solution was cross‐linked at temperatures 4°C, 20°C and 37°C with time intervals of 6, 12, 18 and 24 hours. For biological tests of the composite materials in vitro and in vivo, the mixed solution was cross‐linked at 20°C for 24 hours. The cross‐linked composites were frozen at −20°C for 24 hours, followed by lyophilization for 24 hours. The scaffolds were then neutralized with 0.1 M Na_2_HPO_4_, washed with distilled water and freeze‐dried for further use.

### 2.4 Measurement of the TA release from PLGA/TA microspheres and ChCo‐TAMS scaffold

Five milligrams PLGA/TA microspheres or ChCo‐TAMS scaffold loaded with 20 mg microspheres were kept in 5 or 20 mL phosphate buffer solution (PBS) with different pH conditions (5, 6, 7) under constant shaking at 37°C. The solvent was collected for HPLC assay and replaced with fresh pH‐adjusted PBS at desired time points (1, 3, 6, 10, 15, 22, 32, 42, 52, 62, 72, 82 and 92 days).

### 2.5 Characterization of differentially cross‐linked scaffolds

The constructed scaffolds were mounted onto aluminium stubs, coated with gold and scanned using scanning electron microscope (SEM) (JEM‐T300, Jeol Inc, Japan). To measure the swelling ratio, different groups of dry scaffolds were weighed (W_d_) and immersed in PBS. After 24 h, the scaffolds were removed and weighed (W_s_). The swelling ratio was determined as $E_{s}\left(\%\right)=\left(W_{s}‐W_{d}\right)/W_{d}\;\times \;\text{1}00$. To evaluate the degree of cross‐linking, the procedure of determination was performed using the Ninhydrin kit (Wako, Japan). Specifically, a calibration curve was created using various concentrations of the standard glycine substance. The scaffold sample (5 mg) was incubated in 4 mL ninhydrin at 80°C for 15 minutes. The standard substance and samples were cooled and diluted with 60% ethanol, followed by absorbance detection at a wavelength of 570 nm. The mass of free amino acids (W_f_) in the scaffolds was determined based on the calibration line, and its ratio was calculated as $R\;=\;W_{f}/\text{5}\;\text{mg}\;\times \;\text{1}00\%$. The final cross‐linking degree of each scaffold was calculated as follows: $\text{CD}\;=\;\text{1}‐R_{\text{Cross}‐\text{linked}}/R_{\text{Un}‐\text{cross}‐\text{linked}}$. The tensile strength of the scaffolds was measured using a texture analyser (TA.XT2, UK). Briefly, scaffold samples were cut into pieces (10 mm × 50 mm) and fixed into clamps. The moving speed of the clamp was set to 5 mm/min. Tensile strength was evaluated as the breaking force per cross‐sectional area. For degradation assessment of the composite materials, differentially cross‐linked scaffolds were incubated in 0.1 M PBS (pH 7.4) containing 1.5 μg/mL lysozyme and agitated at 37°C with a daily solution replacement. The scaffolds were then freeze‐dried and weighed weekly. The dry weight initially and at the time point tn was termed as (Wi) and (Wtn), respectively, while the degradation rate was calculated as Dr = (Wi‐Wtn)/Wi*100%.

### 2.6 Assay of cell adhesion on ChCo‐based scaffolds

NIH‐3T3 and TE‐1 cells were seeded on ChCo or ChCo‐TAMS scaffolds for 5 days in 12‐well dishes. Then, the scaffolds were fixed and embedded with tissue freezing medium, and sectioned at 10 μm for haematoxylin and eosin (H&E) observation under a microscope. For SEM scanning, the scaffolds were washed with PBS, dehydrated in a series of ethanol and freeze‐dried before being coated with gold for observation.

### 2.7 Determination of cell viability

Cell viability of RAW264.7 or L929 cultured on scaffolds was determined by CCK‐8 (E606335, Sangon Biotech) according to the manufacturer's protocol. Briefly, different composites (1 mL prior to freeze‐drying; ChCo contained 5 mg chitosan and 5 mg collagen, whereas ChCo‐TAMS containing additional 20 mg PLGA/TA microspheres) were added to 1 mL or 2 mL of culture medium (1:1 Dulbecco's modified Eagle's medium to Ham's F‐12 medium supplemented with 10% FBS, 0.224% NaHCO_3_ and 1% penicillin‐streptomycin) after cell seeding at a density of 1 × 10^5^ cells/mL. After culturing for 24 or 48 hours, cell viability was determined at 450 nm using a microplate photometer (Thermo Scientific). Cells were also stained with calcein AM and PI (Life Technology) for 15 minutes at 37°C, rinsed with PBS and then imaged using a fluorescence microscope (Nikon Eclipse Ti‐U, Japan).

### 2.8 Assay of cell migration

Migration of RAW264.7 cells was measured using transwell chambers with 8‐μm pores (Costar, Cambridge, MA). Cells were seeded in the upper chambers at a density of 1 × 10^5^ cells/mL, while the lower chambers contained 1 μg/mL lipopolysaccharide (LPS), in the presence or absence of the composites (10 mg ChCo; 30 mg ChCo‐TAMS containing 10 mg ChCo and 20 mg PLGA/TA microspheres) or 1.2 mg of TA in 1 mL culture medium. The cells were allowed to migrate for 24 hours, followed by staining with crystal violet and microscopic observation. The stained cells were calculated using the ImageJ software. For cellular chemotaxis evaluation, cells were seeded in the lower chambers, with or without LPS stimulation, in the presence or absence of the composites or TA in 1 mL culture medium. After 24 hours of culture, the medium in the lower chambers was replaced, and untreated cells were seeded in the upper chambers. After another 24 hours of incubation, the migrated cells were stained and counted.

### 2.9 ELISA

RAW264.7 cells were cultured in 1 mL DMEM medium containing different composites as described above or 1.2 mg of TA in the presence of 1 μg/mL LPS (Sigma) for 24 hours. The morphology of the cells was observed under an optical microscope and quantified using ImageJ software at high power field (HPF). Cell supernatants were harvested, and cytokines, including TGF‐β1, TNF‐α and IL‐6, were detected using ELISA kits (BD Biosciences, R&D Systems) according to the manufacturer's instructions.

### 2.10 Western blot

Cells following the desired treatment were lysed using RIPA lysis buffer (Beyotime, China) containing protease and phosphatase inhibitors. Protein extracts were heat‐denatured at 95°C for 5 minutes, electrophoretically separated on 10% SDS‐PAGE and transferred to 0.22 μm PVDF membranes (Sigma). The membranes were then incubated with primary antibodies at 4°C overnight, washed and probed with HRP‐conjugated secondary antibodies at room temperature for 2 hours. GAPDH was used as an internal control. The primary antibodies used in Western blotting were as follows: PCNA (sc‐56, Santa Cruz), Bax (A19684, Abclonal), Cleaved‐Caspase3 (9664, CST), Caspase3 (9662, CST), GAPDH (60004‐1‐Ig, Proteintech), α‐SMA (A1011, Abclonal), Collagen‐I (A1352, Abclonal), MMP2 (40 994, CST), MMP9 (13 667, CST), P‐Smad2 (26 945, CST), Smad2 (5339, CST).

### 2.11 Quantitative real‐time PCR

Cells were collected and lysed using TRIzol. Extraction of total RNA and reverse transcription of cDNA were performed using kits according to the manufacturer's instructions. qPCR was performed on a LightCycler480II (Roche) system with SYBR green mix kit (Vazyme). The data were presented using the comparative C_t_ method and statistically analysed using GraphPad Prism7. The relative values of all the results were normalized to those of the control. The primers for amplification were listed as follow: TGFβ1, F: 5′‐CCA CCT GCA AGA CCA TCG AC‐3′, R: 5′‐CTG GCG AGC CTT AGT TTG GAC‐3′; IL‐6, F: 5′‐CTG CAA GAG ACT TCC ATC CAG‐3′, R: 5′‐AGT GGT ATA GAC AGG TCT GTT GG‐3′; TNF‐α, F: 5′‐CAG GCG GTG CCT ATG TCT C‐3′, R: 5′‐CGA TCA CCC CGA AGT TCA GTA G‐3′; α‐SMA, F: 5′‐CCC AGA CAT CAG GGA GTA ATG G‐3′, R: 5′‐TCT ATC GGA TAC TTC AGC GTC A‐3′; Collagen‐I, F: 5′‐GCT CCT CTT AGG GGC CAC T‐3′, R: 5′‐ATT GGG GAC CCT TAG GCC AT‐3′; Collagen‐III, F: 5′‐CTG TAA CAT GGA AAC TGG GGA AA‐3′, R: 5′‐CCA TAG CTG AAC TGA AAA CCA CC‐3′; MMP2, F: 5′‐ACC TGA ACA CTT TCT ATG GCT G‐3′, R: 5′‐CTT CCG CAT GGT CTC GAT G‐3′; MMP9, F: 5′‐GCA GAG GCA TAC TTG TAC CG‐3′, R: 5′‐TGA TGT TAT GAT GGT CCC ACT TG‐3′; GAPDH, F: 5′‐AGG TCG GTG TGA ACG GAT TTG‐3′, R: 5′‐GGG GTC GTT GAT GGC AAC A‐3′.

### 2.12 Determination of ChCo‐based scaffold effects on fibrosis in vitro

L929 cells and NIH3T3 cells were cultured in 1 mL DMEM medium containing the composites or TA as described above for 24 h or 48 hours, in the presence of 10 ng/mL of mouse recombinant TGFβ1 (Meilun Biotechnology, China). Subsequently, the cells were lysed and collected for RT‐PCR and immunoblotting assays. Alternatively, NIH3T3 cells were fixed with 4% formaldehyde, permeabilized with 0.25% Triton X‐100 and then blocked with 1% BSA for 1 hour at room temperature. They were further incubated with α‐SMA (Abclonal) or FAP (Cell Signaling Technology) antibodies diluted with 1% BSA. After washing with PBS, the cells were conjugated with the fluor‐labelled secondary antibodies (ABclonal Technology) or DAPI (CST), and the mean fluorescence intensity values were determined using ImageJ software. To determine the effects of ChCo‐based scaffolds on inflammation‐associated fibrogenesis, RAW264.7 cells were induced with 1 μg/mL of LPS in the presence or absence of the composites or TA for 24 hours. The conditioned culture medium was then collected to stimulate L929 cells for 24 hours. The control groups of L929 cells were stimulated with the same concentration of LPS. Immunoblotting was performed to assess alterations in fibrosis‐related proteins.

### 2.13 Functional evaluations of ChCo‐based scaffolds in the rat dermal defect model

The 8‐week male Sprague‐Dawley (SD) rats were grouped as Control, ChCo, ChCo‐TAMS and TA treatment with 8 rats each. Approximately 1.5 × 1.5 cm^2^ of dorsal skin of the rat was resected after the animals were anaesthetized with an intraperitoneal injection of 10% chloral hydrate (3 mg/kg), and the fur was shaved from the surgical site. The ChCo‐based scaffolds were attached to the injured sites, or TA (1.2 mg) was subcutaneously injected prior to gauze fixation. The same dose of TA was repeatedly injected in the following week. The wound healing of the animals was recorded weekly. The fibrotic thickness of the skin in each sample was measured and quantified. The percentage of contractile area was calculated as follows: Area (%) = (initial measured wound area − final scar area)/initial wound area. At the first and 6th weeks, three rats from each group were sacrificed and the healing skin in situ was collected for H&E, immunohistochemistry, Masson's trichrome and Sirius Red staining. The antibodies used in immunohistochemistry were TGFβ1 (Sc130148, Santa Cruz), α‐SMA (A1011, Abclonal), Collagen‐I (A1352, Abclonal), Fibronectin (A12932, Abclonal), CD68 (ab125212, Abcam) and MPO (ab208670, Abcam). Masson's trichrome and Sirius Red staining were performed according to the manufacturer’s protocols (Masson's trichrome staining kit, Sigma; Sirius Red staining kit, Senbeijia biotechnology). The studies were approved by Animal Ethics Committee of Nantong University.

### 2.14 Functional evaluations of ChCo‐based scaffolds in the porcine oesophageal ESD model

Four white pigs aged 3 months from Shanghai were anaesthetized and subjected to tracheal intubation and circumferential oesophageal ESD. Two of the pigs were implanted with a bare oesophageal stent (Nanjing Micro‐Tech Endoscopy), and the others were implanted with stents wrapped with ChCo‐TAMS. The ChCo‐TAMS composed of 50 mg chitosan, 50 mg collagen‐I and 200 mg PLGA/TA microspheres was made into a cylindrical membrane with an inner diameter of 2 cm, a thickness of 2 mm and a permeability of 7.8 × 10^−10^ m^2^. The permeability was estimated according to the Kozeny‐Carmen‐based equation ($\text{k =}\;\frac{\pi }{\text{128}}{\;n}_{A}d^{\text{4}}$), where k is the permeability (m^2^), n_A_ is the number of pores per unit area, and d is the pore diameter. n_A_ (975 ± 82 per mm^2^) and d (75.6 ± 16.7 μm) were determined according to previous SEM data. The stents and wrapped scaffolds were fully covered in the wound bed. Wound healing was observed every ten days. The studies were approved by Animal Ethics Committee of Zhongshan Hospital.

### 2.15 Statistical analysis

Data from three independent experiments were analysed with GraphPad Prism7 software by one‐way ANOVA with Dunnett's post hoc test, and results were considered statistically significant at *P* < 0.05.

## 3 RESULTS

### 3.1 Conditional optimization of genipin cross‐linking for construction of porous chitosan/collagen scaffolds

**FIGURE 1:**
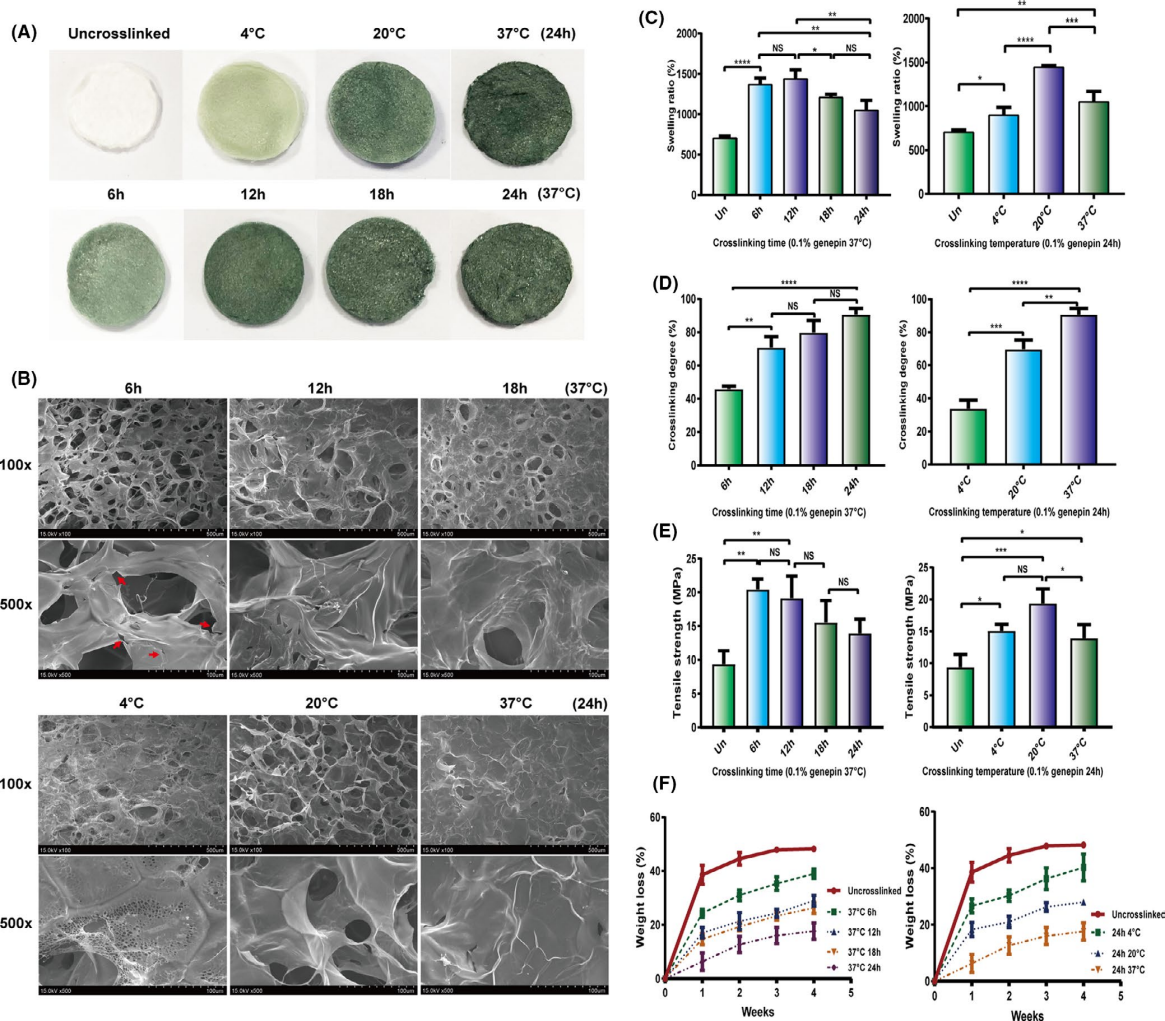
Optimization for differentially cross‐linked porous ChCo scaffolds (A) The colour changes of the porous scaffolds according to different genipin cross‐linking. (B) The surface structure of differentially cross‐linked scaffolds identified by SEM; the red arrows indicated the fissures of the pore wall. (C‐F) The swelling ratio, cross‐linking degree, tensile strength and degradation rate alterations of differentially cross‐linked scaffolds. The representative statistical results were performed using one‐way ANOVA test and shown as means ± SEM from 3 independent experiments. *P < 0.05, **P < 0.01, ***P < 0.001, ****P < 0.0001

To construct stable porous ChCo scaffolds, genipin was used as a cross‐linker to produce proper composites comprising chitosan and collagen. As 0.1% of genipin has been shown to have minimal cytotoxicity,^20^ porous ChCo scaffolds were fabricated at different temperatures and reaction times following the addition of 0.1% genipin. The results demonstrated that the colour changes of the composites, as reflected by the reacted amino acids, significantly increased with an increase in the temperature or reaction duration (Figure 1A). The cross‐linking degree by evaluating the consumed amino groups in the cross‐linked scaffolds showed a consistent conclusion, with a peak value at reaction conditions of 37°C for 24 hours (Figure 1D). Observation of the surface morphology of the cross‐linked scaffolds by SEM revealed that reticular structures with irregularly interconnected pores were formed in the composites, and the walls of the pores gradually fused in a duration‐dependent manner at 37°C, with a completely compact appearance at 24 hours. In another aspect, temperature also mattered, as cross‐linking at 20°C for 24 hours manifested a more stable structure than either at 4°C or 37°C (Figure 1B). In conclusion, scaffolds cross‐linked at 20°C for 24 hours produced preferable structures with smooth pore walls and fewer wall fissures, which are biomimetic for cell adhesion and growth. To test the correlations between reaction conditions and other properties of the ChCo composites, swelling ratio involving in water retention and substances exchange was then measured. The data showed that the swelling ratio of the materials increased with the extension of reaction time from 6 hours and decreased from 18 to 24 hours at 37°C. In addition, the parameter also increased with an increase in temperature from 4 to 20°C and decreased at 37°C for 24 hours (Figure 1C). The mechanical properties of the synthesized materials were determined. The results demonstrated that the optimal reaction conditions for the tensile strength were at 37°C for 6 and 12 hours, or at 20°C for 24 hours (Figure 1E). The data from assays of degradation rate using 1.5 μg/mL lysozyme showed that the ChCo composites reduced weight loss in a cross‐linking time‐ and temperature‐dependent manner, in comparison with the un‐cross‐linked scaffolds containing 50% chitosan (Figure 1F). Taken together, the porous ChCo scaffolds synthesized at 20°C for 24 hours exhibited favourable properties in terms of the proper spatial structure, moderate cross‐linking degree, swelling ratio, tensile strength and low degradation.

### 3.2 Fabrication and determination of drug release of PLGA/TA microspheres and porous ChCo‐TAMS scaffold

**FIGURE 2:**
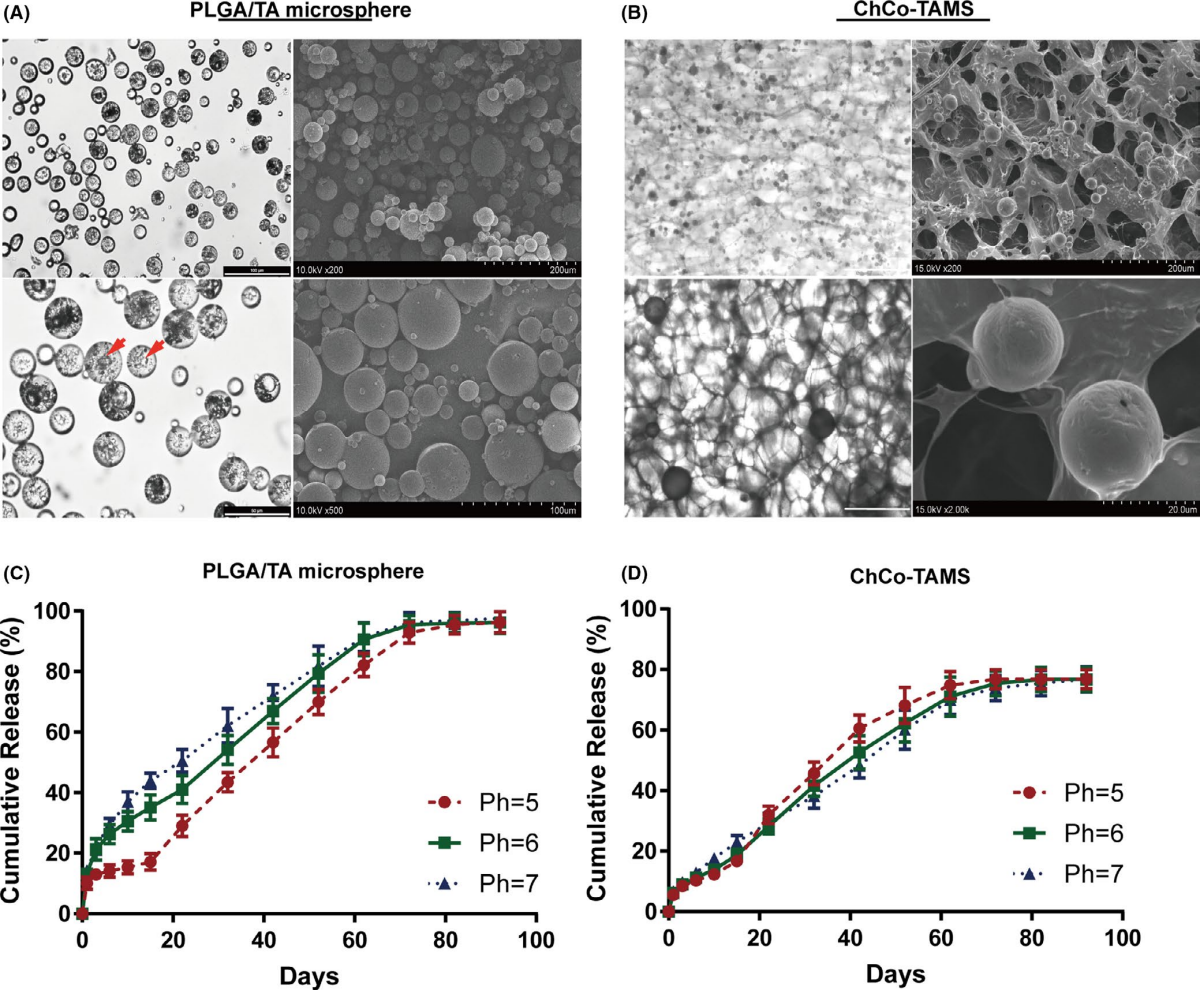
Preparation of PLGA/TA microspheres, ChCo‐TAMS and evaluation of the drug release in vitro (A) Morphological observations of the prepared PLGA/TA microspheres via optical microscopy and SEM scanning; the red arrows indicated the encapsulated TA particles. (B) Morphological observations of the ChCo‐TAMS scaffolds via optical microscopy and SEM scanning. (C, D) The long‐term release of TA from PLGA/TA microspheres or ChCo‐TAMS at various PH conditions in vitro

By method of solid‐in‐oil‐in‐water (s/o/w) double‐emulsion solvent evaporation, PLGA/TA microspheres were successfully prepared with a theoretical drug loading of 10%, drug loading of 5.79 ± 0.22%, encapsulation efficiency at 57.9 ± 2.2% and particle size at 25.53 ± 4.28 μm. Light microscopy and SEM scanning also revealed that TA was encapsulated in these composite microspheres, indicating a potential microscale release system (Figure 2A). To combine the PLGA/TA microspheres with ChCo scaffolds, 20 mg of PLGA/TA microspheres was added to the ChCo composites (1 mL compound solution containing 5 mg chitosan and 5 mg collagen) and cross‐linked with 0.1% genipin at 20°C for 24 hours. Thus, porous ChCo‐TAMS scaffolds were fabricated with a fibrillary network structure firmly attached to the PLGA/TA microspheres (Figure 2B). To evaluate the efficiency of drug release from the microspheres or ChCo‐TAMS scaffolds, TA from the solvent was determined by HPLC after 5 mg PLGA/TA microspheres or ChCo‐TAMS scaffold loading 20 mg microspheres were dissolved in 5 ml or 20 ml PBS at 37°C under physiological oesophageal pH conditions ranging from 5.0 to 7.0. The results showed that TA released from PLGA/TA microspheres could be assayed for up to 90 days. However, changes in pH value were able to moderately affect the drug release, in that high pH conditions promoted the release, especially during the first 20 days (Figure 2C). Distinctively, once the PLGA/TA microspheres were incorporated into the ChCo scaffolds, the release of TA was hardly influenced by pH changes. In addition, the drug release rate was reduced by 20% compared to that of unattached PLGA/TA microspheres (Figure 2D). These data indicate that the ChCo‐TAMS scaffolds improved the stability of TA release.

### 3.3 Characterization of ChCo‐based scaffolds for cell adhesion and viability

**FIGURE 3:**
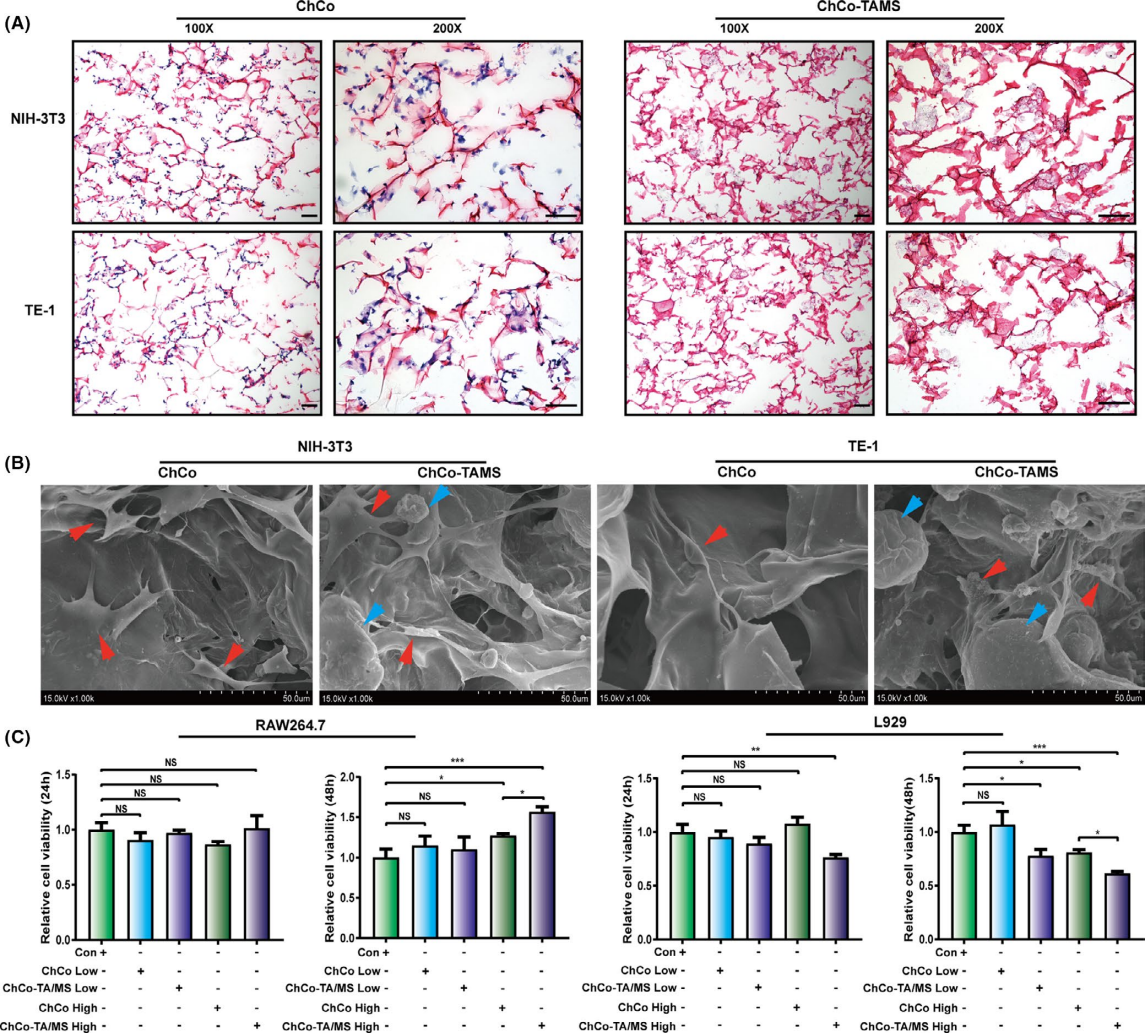
Determination of the cell adhesion and viability on ChCo‐based scaffolds (A) NIH‐3T3 and TE‐1 cells were seeded in the ChCo‐based scaffolds for 5 days, followed by H&E staining; scale bar, 100 μm. (B) Cells attached scaffolds were evaluated by SEM; the red arrows indicated the adhered cells, and blue arrows indicated the attached PLGA/TA microspheres. (C) Identification of the cell viabilities of RAW264.7 and L929 in the presence of the ChCo‐based scaffolds at different concentrations and indicated time intervals. The representative statistical results were performed using one‐way ANOVA test and shown as means ± SEM from 3 independent experiments. *P < 0.05, **P < 0.01, ***P < 0.001, ****P < 0.0001

To elucidate the effects of the ChCo‐TAMS scaffolds on cell adhesion and viability in oesophageal mucosa and submucosal tissues, cell lines including NIH‐3T3, L929 (fibroblasts), TE‐1 (oesophageal epithelial cells) and RAW264.7 (macrophages) were co‐cultured with ChCo and ChCo‐TAMS scaffolds. H&E staining and SEM scanning revealed that both scaffolds fulfilled the adhesion of fibroblasts (NIH‐3T3) and epithelial cells (TE‐1) by internal attachment with a natural fibroblastic morphology and spread pseudopodia (Figure 3A,B). As macrophages and fibroblasts are important contributors to fibrogenesis, it is necessary to know whether the composites influence the viability of the two cell types. Thus, the same density of RAW264.7 and L929 was cultured in 1 or 2 mL DMEM containing 10 mg ChCo or 30 mg ChCo‐TAMS (10mg ChCo attached with 20 mg PLGA/TA microspheres) scaffolds. CCK‐8 assays showed that high concentrations of ChCo or ChCo‐TAMS composites increased the survival of macrophages, whereas low concentrations did not affect cell survival after culture for 48 hours (Figure 3C). Comparatively, ChCo‐TAMS was more efficient than ChCo scaffolds in mediating the viability of RAW264.7 cells. As for their influence on another cell line L929, it is interesting to note that the cell viability of L929 was significantly inhibited following culturing with a high concentration of ChCo‐TAMS composites for 24 hours, or at different concentrations with an incubation time of 48 hours. In the context of absent PLGA/TA microspheres, only high concentration of the composites played inhibitory function on the L929 viability at 48 hours (Figure 3C). To observe the cellular effects of the composites at the molecular level, cells were co‐cultured with high concentrations of ChCo or ChCo‐TAMS composites for 48 hours. Calcein/PI staining revealed that both ChCo and ChCo‐TAMS composites remarkably increased the calcein‐positive but reduced PI‐positive cell numbers in RAW 264.7, with a more prominent action of ChCo‐TAMS (Figure S1A,B). Western blot analysis also confirmed that protein levels of proliferation‐related PCNA were significantly increased, while pro‐apoptotic cleaved‐Caspase3 and BAX were decreased (Figure S1C). In contrast, both live and dead L929 cells were reduced after cell culturing in these two scaffolds (Figure S1D,E). Examination of PCNA, cleaved‐Caspase3 and BAX showed a descending tendency (Figure S1F). These data indicate that ChCo and ChCo‐TAMS composites are pluripotent in suppressing the proliferation of fibroblasts but promoting that of macrophages without inducing cell apoptosis.

### 3.4 ChCo‐based scaffolds were able to inhibit inflammatory activation of macrophages induced by LPS

**FIGURE 4:**
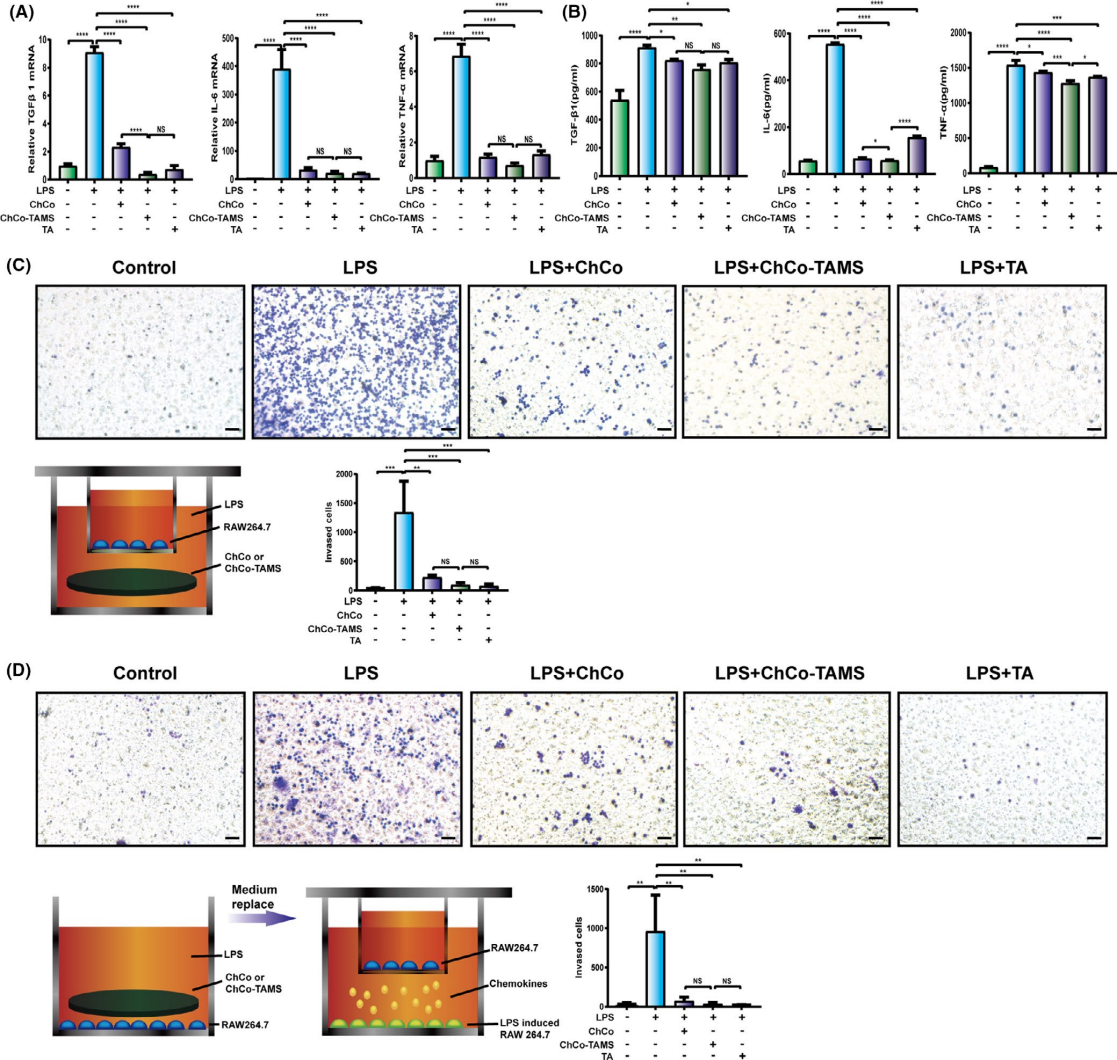
The anti‐inflammatory properties of the ChCo‐based scaffolds in vitro (A) RAW264.7 cells were induced by 1 μg/ml LPS, in the presence of ChCo‐based scaffolds or TA (1.2 mg) in 1 ml medium for 24 h. The mRNA abundance of TGFβ1, IL‐6 and TNFα in different groups was determined by real‐time PCR. (B) The secretion of TGFβ1, IL‐6 and TNFα in different groups was determined by Elisa. (C) and (D) Cellular invasiveness and recruitment of RAW264.7 according to LPS induction and addition of ChCo‐based scaffolds or TA were assessed by transwell assays; scale bar, 50 μm. The representative statistical results were performed using one‐way ANOVA test and shown as means ± SEM from 3 independent experiments. *P < 0.05, **P < 0.01, ***P < 0.001, ****P < 0.0001

Inflammatory cells are immediately recruited into the injured sites to trigger inflammation and eventually mediate fibrosis formation.^21^ Pro‐fibrotic and inflammatory mediators, including TGF‐β1, TNF‐α and IL‐6, which are induced and secreted from activated macrophages, play roles in the transdifferentiation of fibroblasts to myofibroblasts.^22^, ^23^, ^24^ To understand the potential effects of the composites on the inflammatory activation of macrophages, RAW264.7 cells were cultured in 1 mL DMEM containing the composites as described above or 1.2 mg of TA (equivalent drug amount as in ChCo‐TAMS) in the presence of 1 μg/mL of LPS for 24 hours. Light microscopy showed that the cells changed their morphology from sphere‐like to irregular status with extending tentacles following LPS stimulation (Figure S2), while a coculture with ChCo, ChCo‐TAMS or TA showed fewer morphological changes (Figure S2). Further investigation of inflammatory cytokine production and secretion from macrophages demonstrated that the induction of TGF‐β1, TNF‐α and IL‐6 by LPS was significantly suppressed by the addition of ChCo, ChCo‐TAMS or TA, as examined by RT‐PCR and ELISA (Figure 4A,B). Subsequent transwell assays showed that LPS‐mediated cell invasiveness was abrogated by the composites or TA (Figure 4C). It was known that the activated macrophages were able to recruit quiescent ones via chemotaxis.^25^ To address whether the ChCo‐based scaffolds and TA were able to block the effects of activated macrophages, cells in the lower chambers were stimulated with 1 μg/mL of LPS for 24 hours with or without the presence of the scaffolds or the drug. Then, the medium was replaced with the normal medium, followed by simultaneous seeding of quiescent cells in the upper chambers. After another 24 hours of incubation, LPS‐induced recruitment of cells was sharply abrogated when activated cells were treated with ChCo‐based scaffolds and TA, suggesting an anti‐chemotaxis of the composites (Figure 4D). These data provide evidence that ChCo‐based scaffolds can inhibit the activation of macrophages in vitro.

### 3.5 ChCo‐based scaffolds showed anti‐fibrotic effects in vitro

**FIGURE 5:**
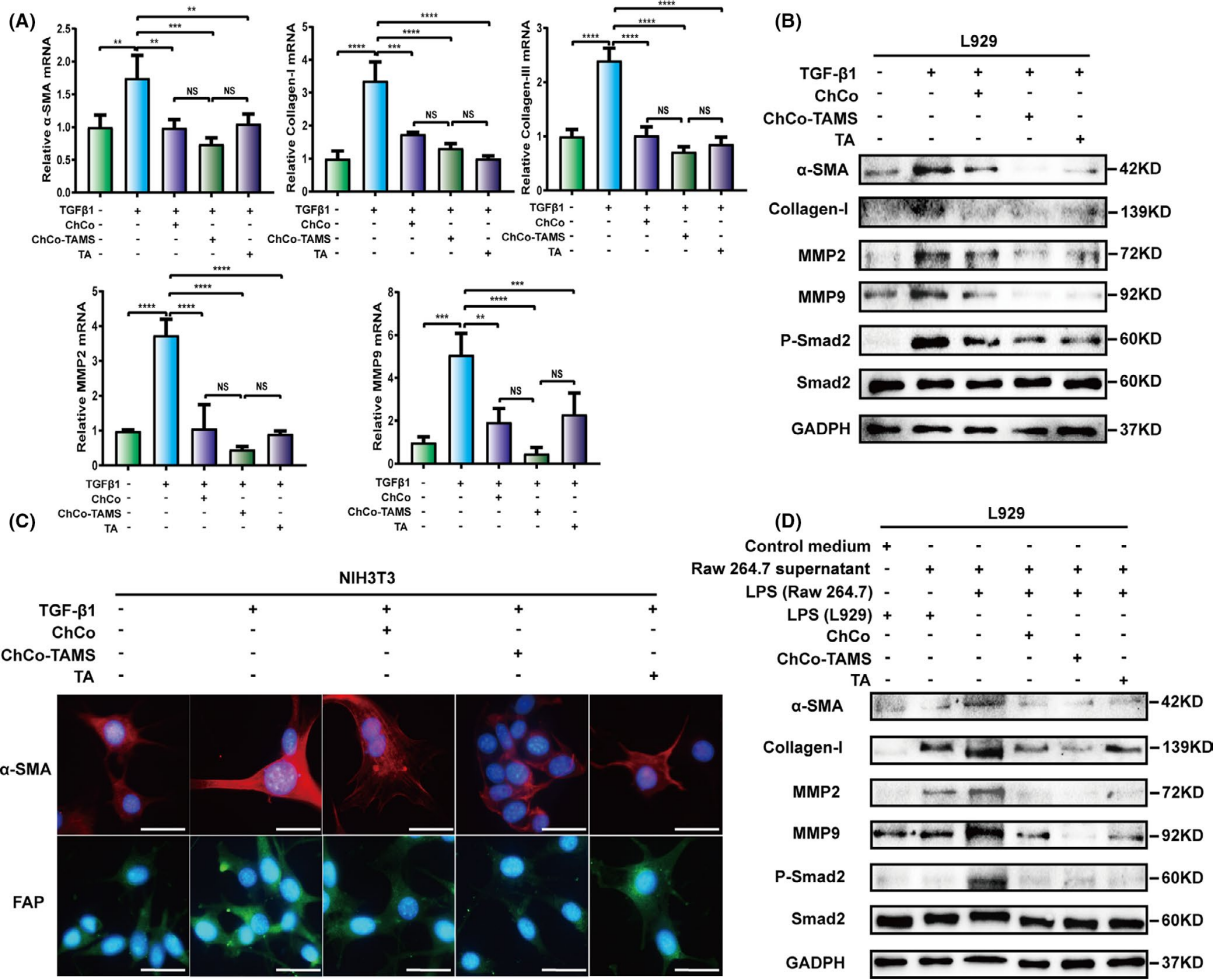
The anti‐fibrogenic effects of the ChCo‐based scaffolds in vitro (A, B) L929 cells were precultured with the ChCo‐based scaffolds or TA for 48 h before induced with 10 ng/ml of TGFβ1 for 24 h, followed by mRNA detection of α‐SMA, collagen‐I, collagen‐III, MMP2, MMP9 or protein detection of α‐SMA, collagen‐I, MMP2, MMP9, p‐Smad2 and Smad2. (C) NIH3T3 cells were precultured with the ChCo‐based scaffolds or TA for 48 h before induced with 10 ng/ml of TGFβ1 for 24 h, followed by immunostaining of α‐SMA and FAP; scale bar, 20 μm. (D) The culture medium of RAW264.7 cells induced by 1 μg/ml LPS in the presence of the ChCo‐based scaffolds or TA for 24 h was collected and applied for culturing of L929 cells at 24 h. Immunoblotting was conducted to assess the variations of α‐SMA, collagen‐I, MMP2, MMP9, p‐Smad2 and Smad2. The representative statistical results were performed using one‐way ANOVA test and shown as means ± SEM from 3 independent experiments. *P < 0.05, **P < 0.01, ***P < 0.001, ****P < 0.0001

To ascertain whether the constructed ChCo‐based scaffolds have anti‐fibrotic roles, an in vitro fibrotic model was established using fibroblasts L929. The cells were induced with 10 ng/mL TGFβ1 for 24 hours in 1 mL DMEM medium, in the presence of the composites or 1.2 mg TA. As data shown, the expression levels of α‐SMA, collagen‐I and collagen‐III were significantly elevated upon TGFβ1 induction, and addition of TA, but not of the ChCo‐based composites, attenuated TGFβ1‐induced effects (Figure S3A). However, an extension of the culture time of the composites to 48 hours remarkably inhibited the mRNA abundance of fibrosis‐related genes induced by TGFβ1, such as α‐SMA, collagen‐I, collagen‐III, MMP2 and MMP9 (Figure 5A). Western blotting also confirmed the results at the protein level, together with phosphorylated Smad2, a downstream effector of TGFβ1 (Figure 5B, S3B). Immunostaining with quantification of α‐SMA and fibroblast activation protein (FAP) performed in fibroblasts NIH3T3 clearly reflected similar protein changes (Figure 5C, S3C). It has been shown that ChCo‐based scaffolds are sufficient to inhibit the activation of macrophages under inflammatory stimuli, so these synthesized composites were thought to interfere with macrophage‐mediated fibrogenesis. The culture medium of RAW264.7 cells induced by LPS in the presence of the composites or TA for 24 hours was collected and applied for culture of L929 cells at 24 hours. Determination of α‐SMA, collagen‐I, MMP2, MMP9 and p‐Smad2 in L929 cells showed that addition of the ChCo‐based composites or TA in the culture medium of RAW264.7 cells remarkably reduced the protein levels (Figure 5D). These results indicate that ChCo‐based scaffolds can suppress fibrogenesis in vitro through multiple mechanisms.

### 3.6 ChCo‐based scaffolds played roles of anti‐fibrosis in the rat dermal defect model

**FIGURE 6:**
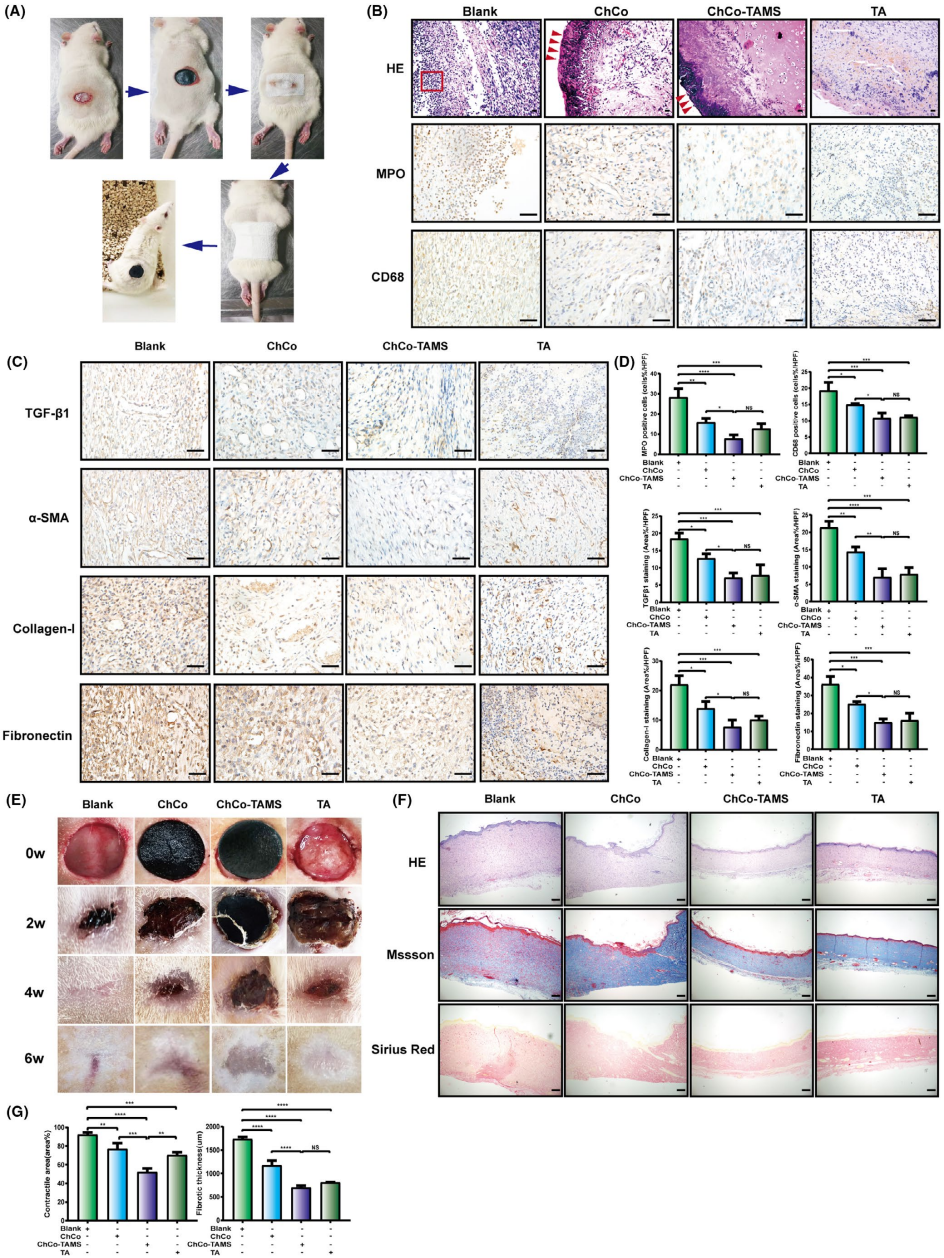
The anti‐fibrotic efficiency of ChCo‐based scaffolds in the rat dermal defect model (A) The procedures of dermal resection and scaffolds fixation on rats. (B) At the 7th day, the wound tissues from different groups were subjected to H&E and IHC staining to evaluate the pathological changes and infiltration of inflammatory cells (MPO‐, CD68‐positive); the red arrows indicated the attached composites and the frame suggested inflammatory cells; scale bar, 50 μm. (C) IHC assays were conducted to identify fibrotic‐related proteins, including TGFβ1, α‐SMA, collagen‐I and fibronectin; scale bar, 50 μm. (D) The quantification of the IHC results. (E) The tissue healing process in different groups was observed at indicated time points. (F) At the 6th week, the scar tissues were collected and assessed by H&E, Masson's trichrome and Sirius Red staining; scale bar, 200 μm. (G) The scar contraction and fibrotic thickness at the 6th week were quantified. The representative statistical results were performed using one‐way ANOVA test and shown as means ± SEM from 3 independent experiments. *P < 0.05, **P < 0.01, ***P < 0.001, ****P < 0.0001

To validate the functions of the constructed ChCo‐based scaffolds in anti‐fibrosis of injured tissues, we first established a rat dermal defect model. Approximate 1.5 cm × 1.5 cm of dorsal skin was resected, followed by the attachment of the scaffolds and gauze fixation. An equivalent of TA (1.2 mg) to the ChCo‐TAMS scaffolds was subcutaneously injected around the lesion sites as a positive control. The scaffolds were observed firmly adhering to the wound bed at 24 hours (Figure 6A), and the healing tissues were collected at 1 week before being subjected to H&E and IHC staining. As presented in Figure 6B, attachment of the ChCo‐based scaffolds to the wound area resulted in less infiltration of inflammatory cells compared to the control. IHC assays revealed that the MPO‐ and CD68‐positive cells were markedly decreased following wound treatment with the scaffolds or the drug (Figure 6B, D). The examination of fibrosis‐related proteins, including TGFβ1, α‐SMA, collagen‐I and fibronectin, demonstrated that ChCo‐based scaffolds and TA administration were capable of inhibiting fibrosis in rat dermal defects. Even not significantly, the ChCo‐TAMS demonstrated the most favourable efficiency (Figure 6C, D). The anti‐fibrotic functions of the composites, together with TA, were also observed in the healing process of the wound. As shown in Figure 6E, application of the ChCo‐based scaffolds and the drug attenuated the contractile effect of the scar, although healing was prolonged until the sixth week (Figure 6E, g). Fibrotic thickness of dermal tissues was evidently thinner than that of the control, as evaluated and quantified by H&E, Masson's trichrome and Sirius Red staining (Figure 6F, g). In conclusion, our data suggest that ChCo‐based scaffolds, especially ChCo‐TAMS, are effective in inhibiting the fibrogenesis of rat dermal defects.

### 3.7 ChCo‐TAMS is efficient in ameliorating ESD‐related oesophageal stricture of swine

**FIGURE 7:**
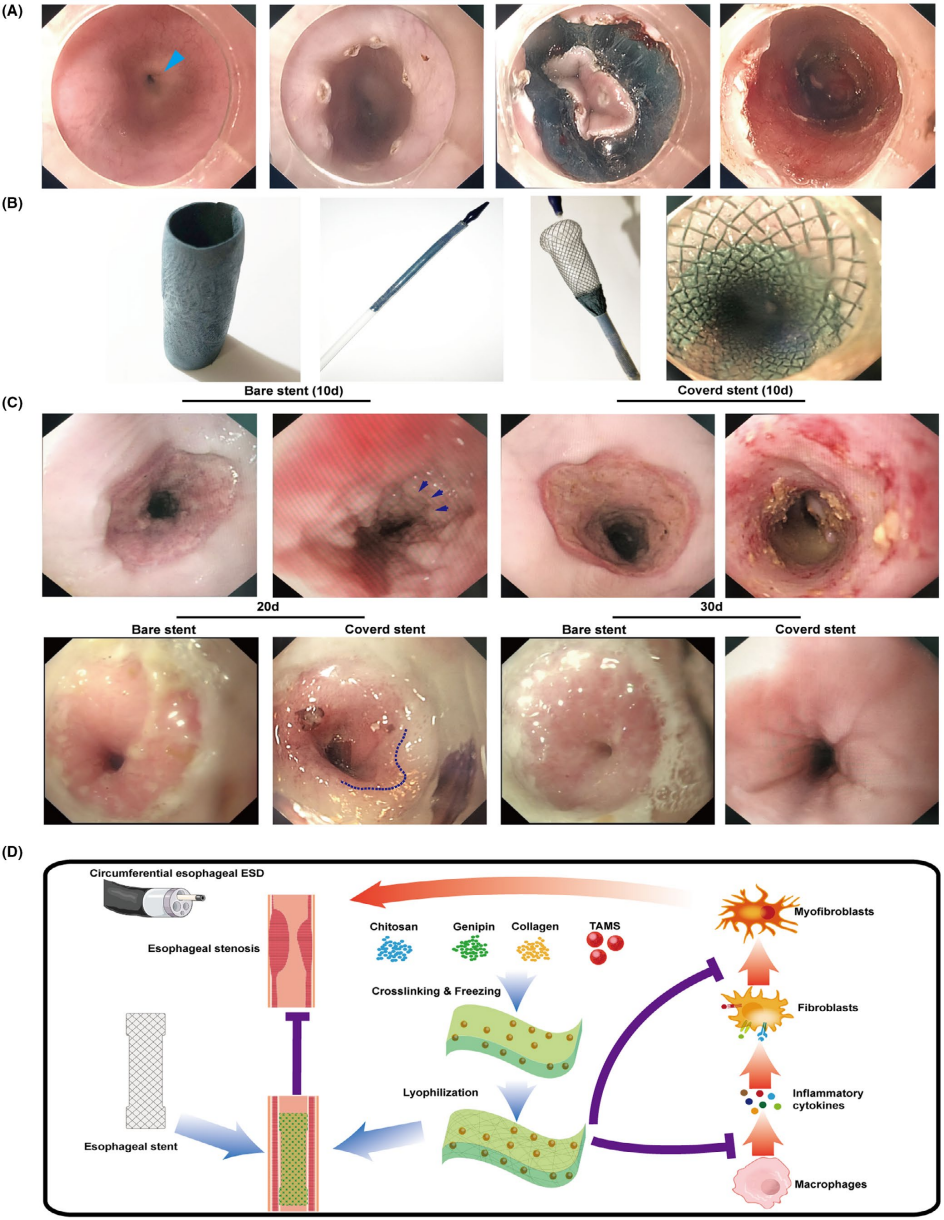
Stricture ameliorating efficiency of ChCo‐TAMS scaffolds in the porcine oesophageal ESD model (A) The circumferential oesophageal ESD procedures. From left to right indicated: normal oesophageal lumen (Blue arrow indicated the cardia); marking of the cutting edge by electrocoagulation; circumferential cutting of the mucosal tissue at the oral side; the post‐operative wound bed. (B) Application of ChCo‐TAMS on post‐ESD wound. From left to right indicated: the prepared cylindrical ChCo‐TAMS; the folded oesophageal metal stent wrapped with ChCo‐TAMS; release of ChCo‐TAMS wrapped stent in vitro; attachment of ChCo‐TAMS onto the post‐ESD wound. (C) Observation of the wound healing with or without the application of ChCo‐TAMS at indicated time intervals. At the 10th day, the arrows indicated the newly regenerated epithelium; at the 20th day, the dotted line indicated the margin between normal mucosa and incompletely healed reddish mucosa. (D) The schematic diagram illustrated the manufacture and the potential mechanisms of the ChCo‐TAMS scaffolds in preventing ESD‐related oesophageal strictures

To examine the effects of the ChCo‐TAMS scaffolds on oesophageal ESD‐related stenosis, a porcine oesophageal ESD model was established for the application of novel biomaterials. Circumferential ESD with a longitude of 3 cm was performed above the cardia (Figure 7A), and a cylindrical ChCo‐TAMS scaffold (approximately 2 mm thickness with a permeability of 7.8 × 10^−10^ m^2^, combined with 50 mg chitosan, 50 mg collagen and 200 mg PLGA/TA microspheres) was wrapped to a mental oesophageal stent. By endoscopic release, the wrapped scaffold was completely attached to the wound bed (Figure 7B). A bare stent was used as a control. The oesophageal stricture began to form 10 days after the operation, with regenerated reticular epithelium in the control group. In contrast, the wound covered by ChCo‐TAMS did not result in stenosis, but also in the absence of newly formed epithelium in the ulcer bed (Figure 7C). These effects were similar to those observed in rat dermal defects. At 20 days after surgery, only a moderate stricture occurred in the ChCo‐TAMS‐treated swine with incomplete healing of the wound, whereas severe stenosis was observed in the control (Figure 7C). The total stricture at a 2 mm diameter was formed at 30 days in the control group. However, ESD surgery supplemented with the ChCo‐TAMS scaffold moderately ameliorated the progression of the oesophageal stricture (Figure 7C). These results indicate that the constructed ChCo‐TAMS scaffolds are efficient in inhibiting ESD‐related oesophageal strictures.

## 4 DISCUSSION

In the last few decades, various approaches based on tissue engineering have been attempted to improve oesophageal post‐ESD stricture, such as autologous epithelial cell sheet transplantation^26^ and oral administration of conditioned medium from mesenchymal stem cells.^27^ Nevertheless, none of them have been successfully applied to clinics because of the limited therapeutic time window, uncertain outcomes and potential safety of the biomaterials. Glucocorticoids are preferentially used in clinics to suppress fibrosis. However, the approach of traditional drug delivery by submucosal injection causes dose loss and risk for perforation. Therefore, a novel running pattern needs to be considered to control oesophageal post‐ESD stricture. In this study, we successfully developed porous composite scaffold by attaching PLGA/TA microspheres, which showed promising application in ameliorating the stricture following ESD.

The properties of the genipin‐cross‐linked scaffolds varied with the alteration of reaction conditions. It was shown that the optimal conditions for the production of ChCo scaffolds were set at cross‐linking for 24 hours at 20°C. Although a long reaction time (24 hours) at high temperature (37°C) resulted in an increased cross‐linking degree with the best degradation resistance, it concomitantly weakened the swelling ratio and mechanical strength. This is probably attributed to the sponginess reduction and structural instability following the pore wall fusion. As for the long‐term release of TA, ChCo‐TAMS scaffolds have an equivalent drug release duration of over 90 days in comparison with the PLGA/TA microspheres. However, the stability of drug release is less affected by changes in pH values, suggesting that ChCo‐TAMS scaffolds are more suitable for application in oesophageal ESD.

In this study, ChCo‐based scaffolds exhibited specific properties in the regulation of cell adhesion, viability and anti‐inflammation/anti‐fibrosis. The composites were found to promote the proliferation and viability of macrophages, whereas they played negative roles in fibroblasts. It can be postulated that the release of TA from the scaffolds may contribute to such distinct effects, as glucocorticoids have been reported to promote proliferation and inhibit apoptosis of macrophages,^28^, ^29^ and reduce both proliferation and apoptosis of fibroblasts.^30^, ^31^ Although the ChCo scaffolds were absent from TA and displayed similar effects to those of ChCo‐TAMS, the most likely reason is attributed to the cross‐linker genipin, which has been proven to have similar anti‐inflammatory and anti‐fibrogenic functions as glucocorticoids.^32^, ^33^ In addition, ChCo‐based scaffolds also acted roles in suppressing LPS‐induced activation of macrophages, chemokine‐mediated cell recruitment and transdifferentiation of fibroblasts, suggesting multiple regulatory mechanisms for cell events.

Both the rat dermal defect and porcine oesophageal ESD model demonstrated that the constructed ChCo‐TAMS scaffolds were efficient in controlling wound contractility or post‐ESD stricture. However, wound healing was delayed when supplemented with the ChCo‐TAMS scaffolds. This is possibly associated with the release of TA, which commonly results in delayed healing as a reaction to anti‐inflammatory drugs. Ongoing studies will focus on the reconstruction of novel composites that will promote wound healing while controlling the stricture of circumferential oesophageal ESD.

In conclusion, as shown in Figure 7D, A novel porous chitosan/collagen scaffold cross‐linked with genipin and attached by PLGA/TA microspheres was successfully constructed, which displayed properties of inhibiting activation of macrophages, chemokine‐mediated cell recruitment and fibrogenesis. Application of the scaffold in the porcine oesophageal ESD model showed its efficiency in preventing post‐operative strictures. Although glucocorticoids are the first line of treatment for stricture prevention during clinical practice, the defects caused by submucosal injection concomitantly result in unsatisfactory outcomes. To this end, the newly designed scaffold precisely ensures the effective drug amount by overcoming the dose leakage of injection, indicating that less endoscopic interventions are required, and may also reduce the risk of local perforation by means of long‐term drug release and muscle layer protection. Taken together, the composite scaffold seems to be an alternative approach for ameliorating post‐ESD stricture and is worthy to be further investigated.

## CONFLICT OF INTEREST

The authors declare no conflict of interest.

## AUTHOR CONTRIBUTIONS

All authors listed contributed to the work. Pinghong Zhou and Wenjie Zheng designed the study. Wenkai Ni, Saiyan Bian and Shengli Lin conducted experiments. Mingbing Xiao and Yongjun Wang wrote and revised the draft. Yumin Yang and Cuihua Lu performed figure artwork. All authors approved final manuscript.

## Supporting information

Figure S1

Figure S2

Figure S3

Figure Legends

## Data Availability

The data that support the findings of this study are available from the corresponding author upon reasonable request.
