# Supplementary figures and images for: Biological testing of chitosan‐collagen‐based porous scaffolds loaded with PLGA/Triamcinolone microspheres for ameliorating endoscopic dissection‐related stenosis in oesophagus
Source: Cell Prolif. 2021 Feb 4;54(3):e13004. doi: 10.1111/cpr.13004 (PMC7941226; doi:10.1111/cpr.13004)

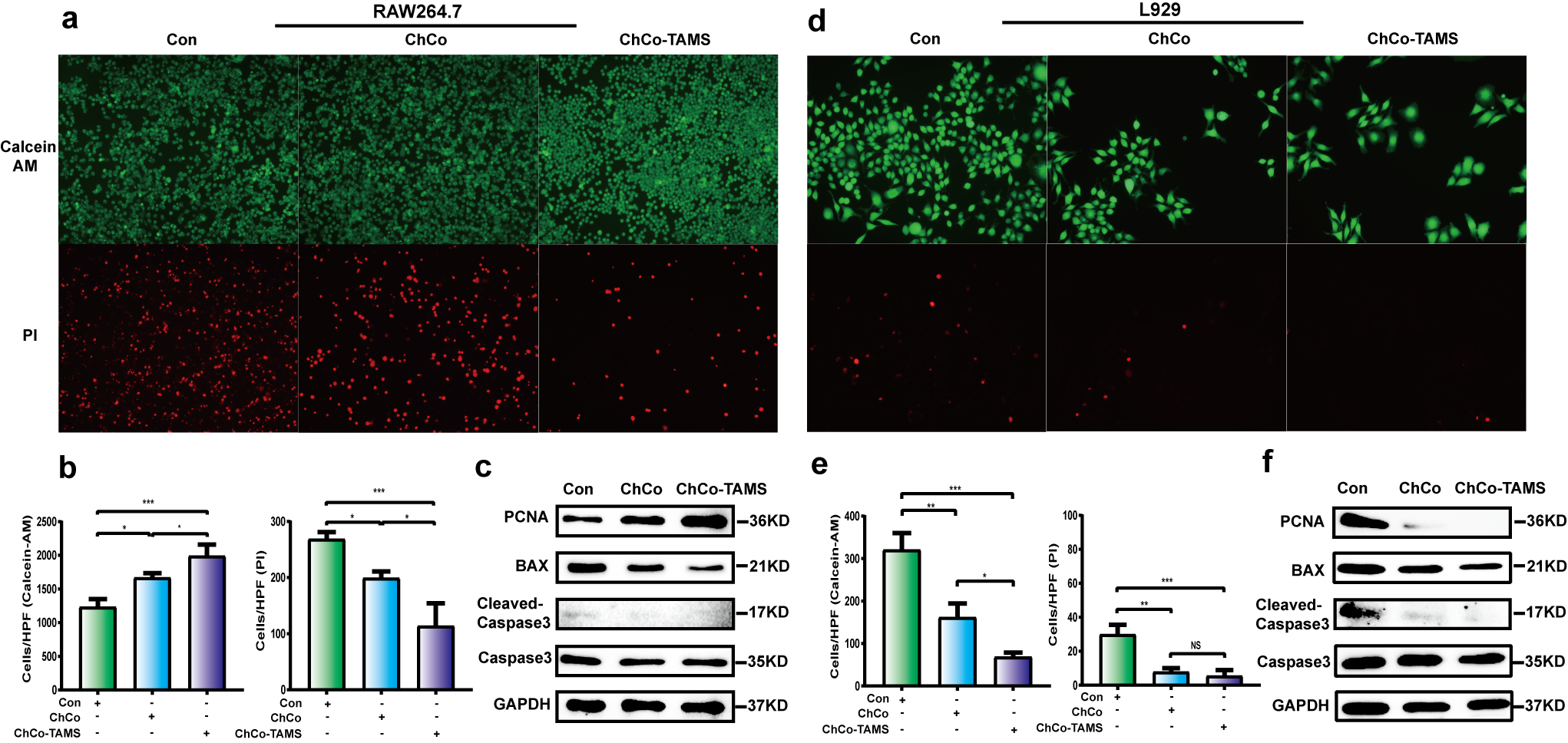

Supplement: Supplementary file 1 — Figure S1 [file CPR-54-e13004-s002.tif]

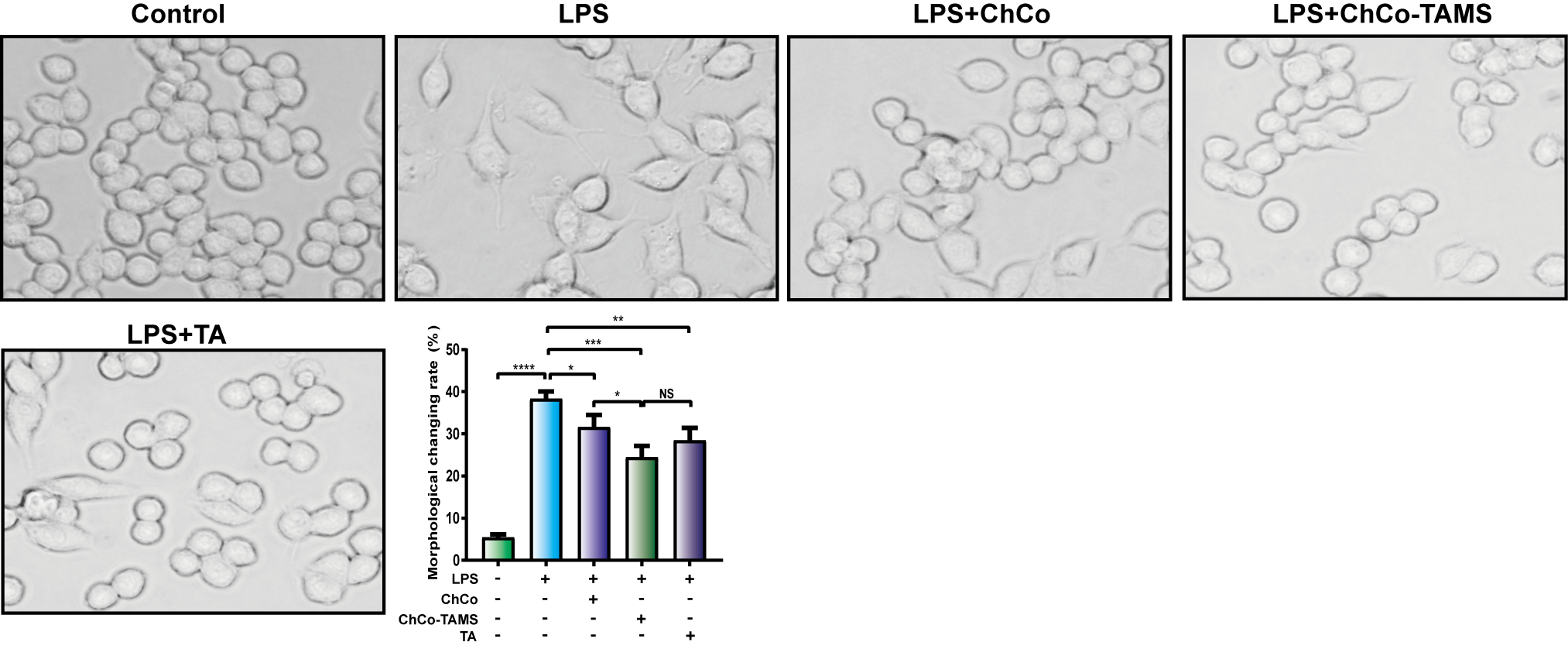

Supplement: Supplementary file 2 — Figure S2 [file CPR-54-e13004-s004.tif]

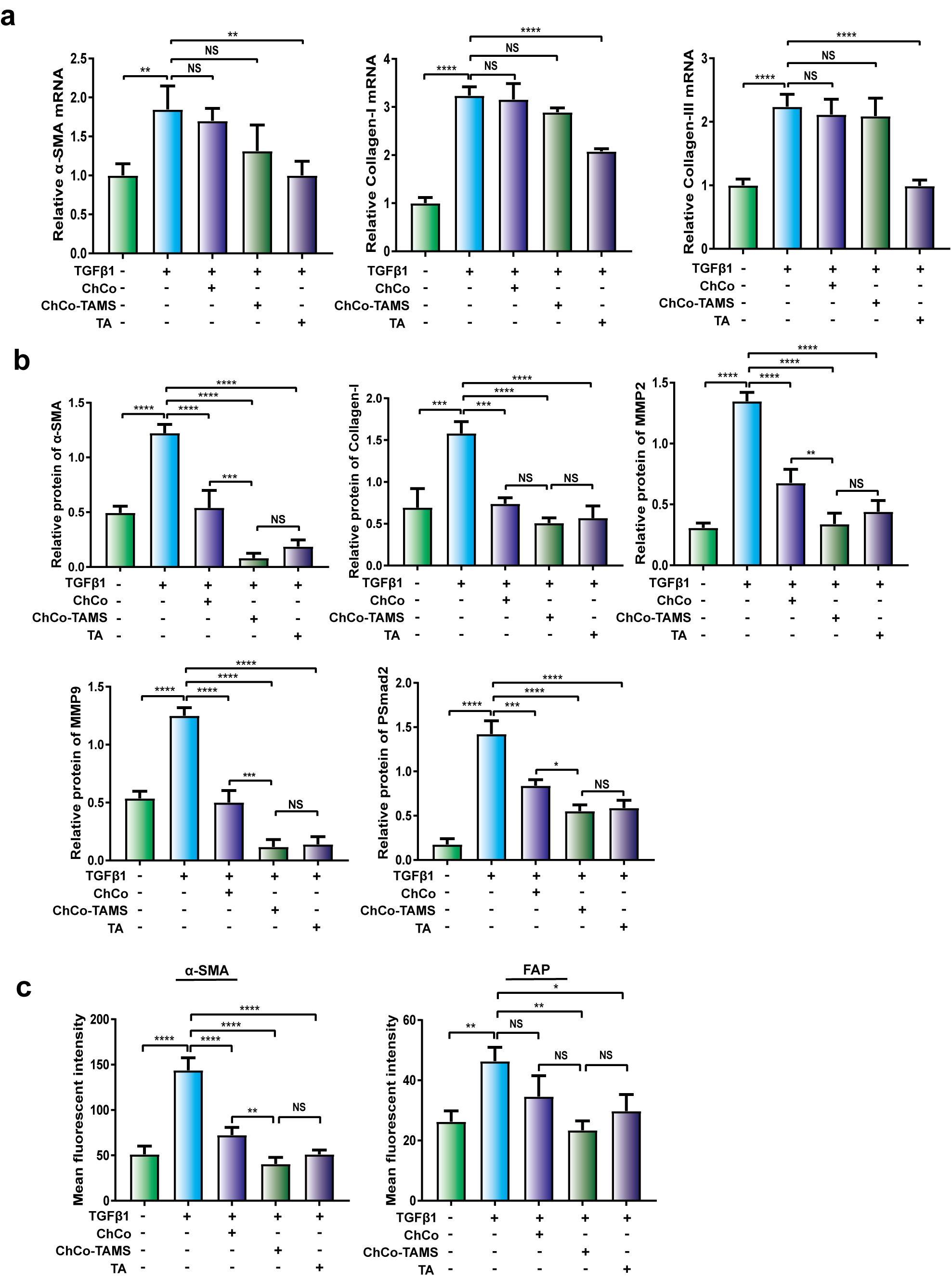

Supplement: Supplementary file 3 — Figure S3 [file CPR-54-e13004-s001.tif]
