# Supplementary material for: Biological testing of chitosan‐collagen‐based porous scaffolds loaded with PLGA/Triamcinolone microspheres for ameliorating endoscopic dissection‐related stenosis in oesophagus
Source: Cell Prolif. 2021 Feb 4;54(3):e13004. doi: 10.1111/cpr.13004 (PMC7941226; doi:10.1111/cpr.13004)
Supplement: Supplementary file 4 — Figure Legends [file CPR-54-e13004-s003.docx]

**Figure S1** (a) (b) RAW264.7 cells were cultured with ChCo-based scaffolds at high concentrations for 48 h, followed by Calcein-AM/PI staining and quantification. (c) Protein alteration of PCNA, BAX, Cleaved-Caspase3, Caspase3 in RAW264.7 cells was determined by immunoblotting. (d) (e) L929 cells were cultured with ChCo-based scaffolds at high concentrations for 48 h, followed by Calcein-AM/PI staining and quantification. (f) Protein alteration of PCNA, BAX, Cleaved-Caspase3, Caspase3 in L929 cells was determined by immunoblotting. The representative statistical results were performed using 1-way ANOVA test and shown as means ± SEM from 3 independent experiments. * p<0.05, ** p<0.01, *** p<0.001, **** p<0.0001.

**Figure S2** RAW264.7 cells were induced by 1 μg/ml LPS, in the presence of ChCo-based scaffolds or TA (1.2mg) in 1 ml medium for 24 h. The morphological alteration of the cells was determined and quantified by ImageJ and GraphPad Prism7 software. The representative statistical results were performed using 1-way ANOVA test and shown as means ± SEM from 3 independent experiments. * p<0.05, ** p<0.01, *** p<0.001, **** p<0.0001.

**Figure S3** (a) L929 cells were induced by 10 ng/ml of TGFβ1 for 24 h at 1 ml DMEM medium, in the presence of the composites or 1.2 mg triamcinolone acetonide. The mRNA abundance of α-SMA, Collagen-I, Collagen-III in each group was determined by real-time PCR. (b) The immunoblotting quantification of Figure 5b. (c) The mean fluorescence intensity quantification of Figure 5c. The representative statistical results were performed using 1-way ANOVA test and shown as means ± SEM from 3 independent experiments. * p<0.05, ** p<0.01, *** p<0.001, **** p<0.0001.
